# Mothers' Care-Seeking Behavior for Common Childhood Illnesses and Its Predictors in Ethiopia: Meta-Analysis

**DOI:** 10.1155/2022/2221618

**Published:** 2022-10-18

**Authors:** Tiwabwork Tekalign, Mistire Teshome Guta, Nefsu Awoke, Abiyot Wolie Asres, Mohammed Suleiman Obsa

**Affiliations:** ^1^School of Nursing, College of Medicine and Health Science, Arba Minch University, Arba Minch, Ethiopia; ^2^School of Nursing, College of Health Science and Medicine, Wolaita Sodo University, Wolaita Sodo, Ethiopia; ^3^School of Public Health, College of Health Science and Medicine, Wolaita Sodo University, Wolaita Sodo, Ethiopia; ^4^School of Anesthesia, College of Health Science and Medicine, Arsi University, Arsi, Ethiopia

## Abstract

**Background:**

Healthcare-seeking interventions can potentially reduce child mortality; however, many children die in developing countries without reaching a health facility. The World Health Organization reported that 70% of child deaths are related to delay care-seeking. So, this review is aimed at identifying mothers' care-seeking behavior for common childhood illnesses and predictors in Ethiopia.

**Methods:**

Systematic search of studies was done on PubMed, Scopus, Web of Science, institutional repositories, Academic Search Premier, and manually from reference lists of identified studies in the English language up to August 2021. The quality of the studies was evaluated by the Joanna Briggs Institute (JBI) quality appraisal tool for prevalence study. This meta-analysis used the random-effect method using the STATA™ Version 14 software.

**Result:**

Fourteen studies involving 8,031 participants were included in this meta-analysis. After correcting Duval and Tweedie's trim and fill analysis, the overall pooled prevalence of mothers' care-seeking behavior is 60.73% (95% CI: 43.49-77.97), whereas the highest prevalence, 74.80% (95% CI: 62.60, 87.00) and 67.77%(95% CI: 55.66, 79.87), was seen in Amhara region and urban residents, respectively, while the lowest, 36.49% (95% CI: -27.21, 100.18) and 47.80% (95% CI:-15.31, 110.9), was seen in South Nation Nationality Peoples' Regions and among rural residents, respectively. Mothers' educational status (*P* ≤ 0.001) and mothers' marital status (*P* ≤ 0.001) were significantly associated with mothers' care-seeking behavior.

**Conclusion:**

Even though children are a vulnerable group, mothers' care-seeking behavior for common childhood illnesses is significantly low. Educational status and marital status were determinants of mothers' care-seeking behavior. So, all responsible bodies should work on the improvement of mothers' care-seeking behavior.

## 1. Introduction

Children represent the at-risk age groups in society. Their mortality rate was cast off as a demographic evaluation and an underlying pointer to the stratum of well-being in uttermost countries: youth troubles and trial disabilities [1]. An estimated 5.4 million children under age 5 stalled in 2017; around half of them went down in sub-Saharan Africa. More than half of these losses were preventable where access to simple and affordable interventions was workable [2].

Likewise, WHO in 2019 reported that a predicted of 5.2 million under five children died; the high prevalence falls on preventable and treatable causes. Children aged 1 to 11 months account for 1.5 million of those deaths and for 1 to 4 years, 1.3 million. Neonate (under 28 days) holds the remaining 2.4 million deaths [3].

Current trends read that close to 48 million children under five will die between 2020 and 2030. Fair half of these under-five deaths will be infants whose deaths can be precluded by attainment high quality gestational care, skilled care at birth, postnatal care, and care of small and sick infants [4].

The World Health Organization (WHO) and United Nations Children's Fund (UNICEF) draw attention to the weight of seeking early care. They developed the Integrated Management of Childhood Illness strategy, highlighting that proper family and community health practices are vital for refining children's health status and declining mortality in developing countries [5]. Causes for the button-down outcome may include issues related to health services, quality of care, health-seeking behaviors, and other factors. Achievement in reducing neonatal mortality necessities is additional than the accessibility of health services with well-trained health professionals and families as the first people responsible for child care [6].

There is a dramatic drop of 60% in under-five mortality from 93 deaths per 1000 live births in 1990 to 38 in 2019. Likewise, in Ethiopia, child mortality rates fell over time. The global burden of child and youth deaths, notwithstanding, remains vast. The burden of child mortality also remains unevenly distributed. The highest under-five mortality rate remains in the WHO African Region (74 per 1000 live births), nine times more developed than that in the WHO European Region (8 per 1000 live births) [7–9].

Care-seeking behavior is a sequence of remedial actions that individuals undertake to rectify perceived ill-health. It is a process by individuals from the time difference between the onset of an illness and getting in contact with a healthcare professional, reasons for the choice of a healthcare professional, and reasons for not seeking help from healthcare professionals [10].

Healthcare-seeking interventions can reduce child mortality; in developing countries, many children die without reaching health institutions. According to a WHO report, 70% of child deaths are due to delayed care-seeking [11, 12].

The home-grounded treatment option is ordinarily exercised for common illnesses by caregivers; different studies showed that limited access to healthcare, high cost of care, and insufficient knowledge about pitfall signs were associated with a lack of care-seeking outside the home [13–16].

Timely and accurate recognition of illness and timely administration of proper treatment by caregivers are critical principles to precluding child deaths [17]. So, this systematic review and meta-analysis play a part in improving mothers' care-seeking behavior for common childhood illnesses in reducing childhood death from preventable and timely treatable illnesses.

### 1.1. Objectives of the Review

- To determine the prevalence of mothers' care-seeking behavior for common childhood illnesses in Ethiopia
- To identify predictors of mothers' care-seeking behavior for common childhood illnesses in Ethiopia

## 2. Methods

### 2.1. Inclusion and Exclusion Criteria

This analysis included published observational studies (i.e., cross-sectional, case-control, and cohort) on mothers' care-seeking behavior for common childhood illnesses in Ethiopia until June 2021. In contrast, studies that did not report overall care-seeking behavior were omitted.

### 2.2. Information Sources, Search Strategy, and Study Selection

The studies were gotten through manual and electronic searches. The databases searched were as follows: PubMed, Scopus, Web of Science, institutional depositories, and manually from reference lists of the former study. Electronic database searching followed by reference lists search used to identify studies, which was also exported into EndNote to remove duplicates. Title and abstract screening was done by authors individually. The Cochrane acronym POCC, which stands for Population, Condition, and Context, was used to decide on all keywords. The keywords used were “childhood” OR “children” OR “under five” OR “neonate” AND “care-seeking” OR “health care seeking “OR “modern care-seeking.” The Preferred Reporting Items for Systematic Reviews and Meta-analyses (PRISMA) statement 2020 guidelines were used to report the findings [18].

### 2.3. Data Collection Process and Data Items

All authors individually extracted the data, and disagreements was elucidated through conversation. A data extraction format was prepared in a Microsoft Excel spreadsheet, containing the name of authors, year of publication, study design, sample size, study area, and the prevalence of care-seeking behavior, and associated factors with their odds ratio were used.

### 2.4. The Outcome of the Review

- The prevalence of care-seeking behavior
- The predictors of care-seeking behavior with their odds ratio

### 2.5. Quality Assessment

All authors independently assessed the quality of each original study using the Joanna Briggs Institute (JBI) quality appraisal tool for prevalence study [19]. If scored 50% or higher, it was considered as low risk.

### 2.6. Data Synthesis and Analysis

A meta-analysis was conducted using the random effect model. The heterogeneity of the studies was assessed using Higgin's *I*^2^ and Cochran's *Q* method. *I*^2^ values of 25% low, 50% moderate, and 75% were considered as high heterogeneity. Also, subgroup analysis was conducted by region and study setting. Additionally, funnel plot and Egger's test were used to check publication bias. For all mentioned analyses, STATA™ V 14 software was used.

## 3. Result

### 3.1. Study Selection

**Figure 1:**
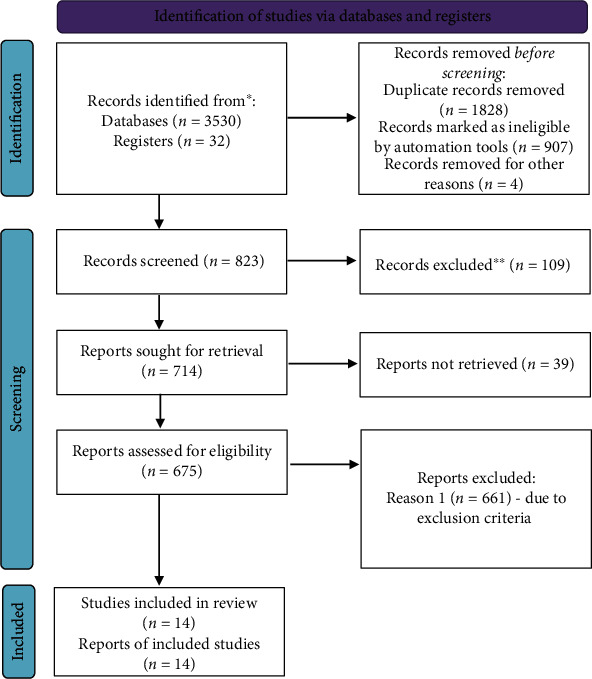
PRISMA flowchart diagram of the study selection.

Initially, 3562 articles were retrieved: 830 from PubMed, 197 from AJOL, 957 from Scopus, 1082 from JSTOR, and 496 from Web of Science databases. Eight hundred twenty-three remained after removing duplicates, then one hundred nine were removed by their title, and thirty-nine articles were not retrieved due to title variation. Then, 675 articles were screened by using the eligibility criteria. Finally, 14 articles met the eligibility criteria and were included in the analysis (Figure 1).

### 3.2. Characteristics of Study

The 14 studies [20–33] included 8,031 participants. Of all, one study is cohort [26], and 13 were cross-sectional. The sample size ranged from 273 [27] to 907 [28]. Most studies were conducted in Amhara and Oromia regions. From the included studies, the prevalence of mothers' care-seeking behavior for common childhood illnesses ranged from 4 [26] to 90.6 [32] (Table 1).

### 3.3. Pooled Prevalence of Care-Seeking Behavior

**Figure 2:**
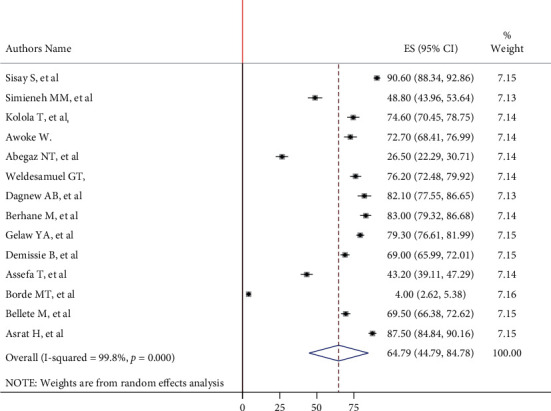
Forest plot showing pooled prevalence of mother's care-seeking behavior for common childhood illnesses in Ethiopia.

The pooled prevalence of care-seeking behavior in Ethiopia, according to this meta-analysis using a random effect model, was 64.78% (95% CI: 44.79, 84.78) with a heterogeneity index (*I*^2^) of 99.8% (*P* ≤ 0.001) (Figure 2). Due to the presence of publication bias which was evident by the Egger test with *P* = 0.002, as a result, the final pooled prevalence was corrected for Duval and Tweedie's trim and fill analysis and was found to be 60.73% (95% CI: 43.49-77.97).

### 3.4. Subgroup Analysis

**Figure 3:**
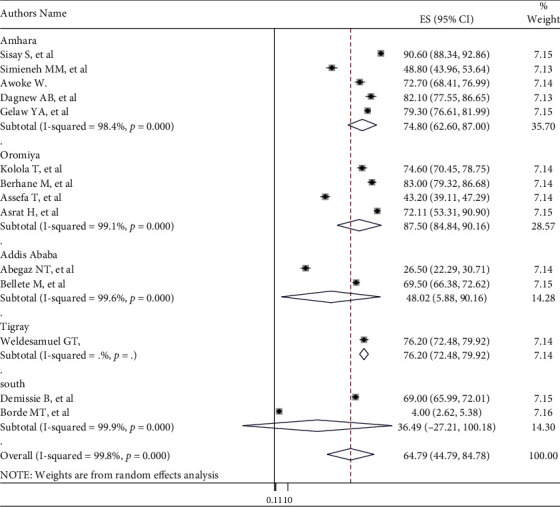
Subgroup analysis of mother's care-seeking behavior for common childhood illnesses in Ethiopia by region.

**Figure 4:**
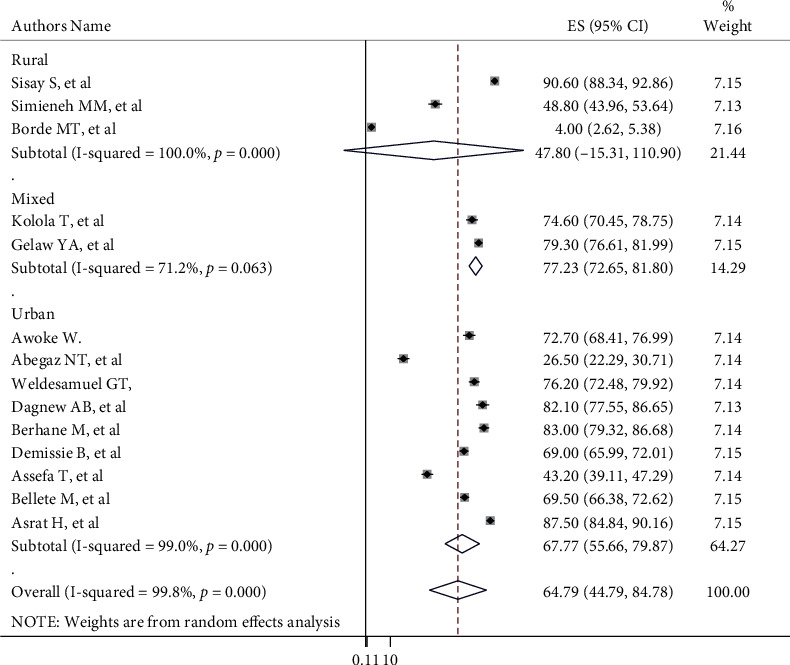
Subgroup analysis of mother's care-seeking behavior for common childhood illnesses in Ethiopia by place of residence.

Subgroup analysis was performed to reduce the likely random variations among studies by considering the prevalence of care-seeking behavior. Accordingly, the highest 74.80% (95% CI: 62.60, 87.00), *I*^2^ = 98.4%) and the lowest 36.49% (95% CI: -27.21, 100.18), *I*^2^ = 99.9%) prevalence was seen in Amhara and South Nation Nationality Peoples' Regions, respectively (Figure 3). Also based on place of residence, the highest 67.77% ((95% CI: 55.66, 79.87), *I*^2^ = 99.0%) and the lowest 47.80% ((95% CI: -15.31, 110.9), *I*^2^ = 100%) care-seeking behavior was seen in urban and rural, respectively (Figure 4).

### 3.5. Metaregression and Sensitivity Analysis

Due to high heterogeneity in subgroup analysis, we have conducted meta-regression to investigate the extent to which statistical heterogeneity between results of multiple studies can be related to one or more characteristics of the studies by using sample size as a covariate but not a significant (Table 2).

### 3.6. Publication Bias

**Figure 5:**
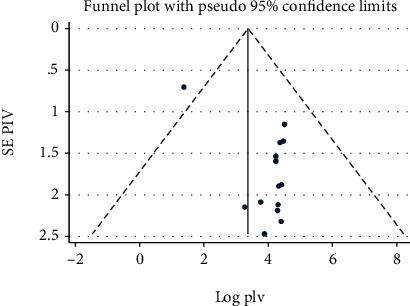
Funnel plot to test the publication bias in 14 studies with 95% confidence limits.

**Figure 6:**
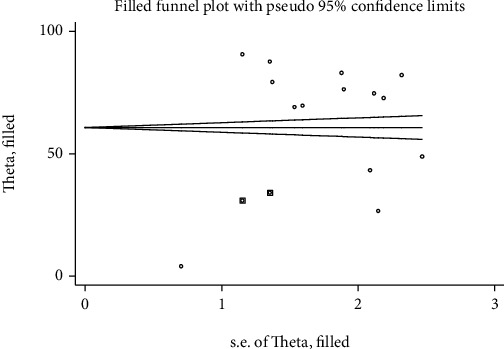
Filled funnel plot after adjusting for publication bias with 95% confidence limits.

The visual inspection of the funnel plot indicated asymmetrical distribution (Figure 5). Additionally, Egger's linear regression test had used to objectively identify publication bias. The result showed that Egger's linear regression test was statistically significant (*t* = 3.82, *P* = 0.003). As a result, it was adjusted for the publication bias by trim and fill analysis; then, the funnel plot appeared symmetrical (Figure 6).

### 3.7. Sensitivity Analysis

**Figure 7:**
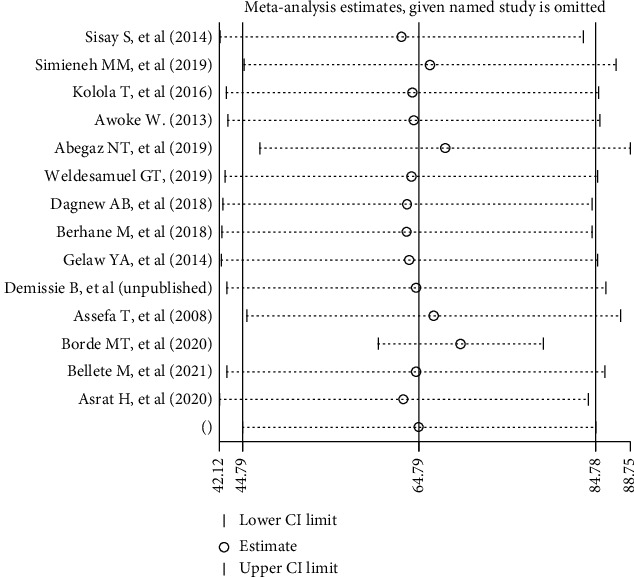
Sensitivity analysis of pooled prevalence for each study being removed one at a time.

Sensitivity analysis result indicated that removing a single study does not significantly influence pooled prevalence (Figure 7).

### 3.8. Predictors of Mothers' Care-Seeking Behavior

Five variables were extracted to identify factors care-seeking behavior for common childhood illnesses. Of all, two variables (educational and marital status) were true predictors (Table 3).

Those mothers not educated were 67% less likely to seek care than educated mothers (OR: 0.33 (95% CI: 0.21-0.54), *P* ≤ 0.001). Single mothers were 65% less likely to seek care than other mothers (OR: 0.35 (95% CI: 0.22-0.55), *P* ≤ 0.001).

## 4. Discussion

Most children in developing countries die from preventable infectious diseases like malaria, diarrhea, and pneumonia; this higher mortality is due to delayed care-seeking behavior and poor access to adequate healthcare [34–36].

According to this study, the overall pooled prevalence of mother's care-seeking behavior after correction by trim and fill analysis was 60.73% (95% CI: 43.49-77.97), which is higher than previous studies conducted in Ethiopia (30%, 29.87%) [37, 38]. Even though most common childhood illnesses are easily manageable due to poor care-seeking behavior, losing those children was common. As one strategy, improving mothers' care-seeking behavior significantly reduces child mortality [39].

The subgroup analysis revealed that the highest prevalence was 74.80%, (95% CI: 62.60, 87.00) seen in Amhara, and the lowest 36.49%; (95% CI: -27.21, 100.18) seen in South Nation Nationality Peoples' Regions; the discrepancy might be due to variation in sociodemographic characteristics and differences in the urbanization level of the regions. Additionally, based on residence, the highest 67.77% (95% CI: 55.66, 79.87) and the lowest 7.80% (95% CI: -15.31, 110.9) healthcare-seeking behavior was seen in urban and rural, respectively. This discrepancy is due to the accessibility of healthcare institutions in settings and sociodemographic features of the mothers.

Additionally, this meta-analysis extracted factors to identify determinants of care-seeking behavior. According to this meta-analysis, educational status has an impact on the care-seeking behavior of mothers. Those mothers not educated were 67% less likely to seek care than educated mothers (OR: 0.33 (95% CI: 0.21-0.54)), which is similar to a study conducted in Bangladesh and Tanzania [40, 41]; evidence shows education has a positive impact on healthcare utilization and care-seeking behavior [42, 43].

Also, the marital status of mothers determines their care-seeking behavior. Single mothers were 65% less likely to seek care than other mothers (OR: 0.35 (95% CI: 0.22-0.55), which is similar to a study conducted in Kenya and Malaysia [44, 45], and this might be because single mothers are worried more about basic needs than immediate medical care.

## 5. Conclusion

Even though children are a vulnerable group, mothers' care-seeking behavior for common childhood illnesses is significantly low. Educational status and marital status were predictors of mothers' care-seeking behavior. So, all responsible bodies should work on the improvement of mothers' care-seeking behavior.

## 6. Limitation of the Study

This systematic review and meta-analysis might have the following limitations. First, due to significant heterogeneity (99.8%), the result should be inferred carefully; secondly, we are unable to compare with other similar studies.

## Figures and Tables

**Table 1:** Characteristics of the included studies in the systematic review and meta-analysis.

Authors' name	Publication year	Study setting	Region	Study design	Sample size	Prevalence (%)	Quality
Abegaz et al. [20]	2019	Addis Ababa	Addis Ababa	Cross-sectional	422	26.5 (22.28-30.71)	Low risk
Asrat et al. [21]	2020	Kimbibit	Oromiya	Cross-sectional	596	87.5 (84.84-90.15)	Low risk
Assefa et al. [22]	2008	Derra	Oromiya	Cross-sectional	563	43.2 (39.10-47.29)	Low risk
Awoke [23]	2013	Bahir Dar	Amhara	Cross-sectional	415	72.7 (68.41-76.98)	Low risk
Bellete et al. [24]	2021	Addis Ababa	Addis Ababa	Cross-sectional	834	69.566.37-72.62)	Low risk
Berhane et al. [25]	2018	Tiro Afeta	Oromiya	Cross-sectional	400	83 (79.31-86.68)	Low risk
Borde et al. [26]	2020	Mekonisa	South	Cohort	772	4 (2.61-5.38)	Low risk
Dagnew et al. [27]	2018	Dangila	Amhara	Cross-sectional	273	82.1 (77.55-86.64)	Low risk
Demissie et al. [28]	2014	Shashogo	South	Cross-sectional	907	69 (65.99-72.00)	Low risk
Gelaw et al. [29]	2014	Bure	Amhara	Cross-sectional	872	79.3 (76.61-81.98)	Low risk
Kolola et al. [30]	2016	Jeldu	Oromiya	Cross-sectional	422	74.6 (70.44-78.75)	Low risk
Simieneh et al. [31]	2019	Aneded	Amhara	Cross-sectional	410	48.8 (43.96-53.63)	Low risk
Sisay et al. [32]	2014	Ansaro	Amhara	Cross-sectional	641	90.6 (88.34-92.85)	Low risk
Weldesamuel et al. [33]	2019	Shire	Tigray	Cross-sectional	504	76.2 (72.48-79.91)	Low risk

**Table 2:** Metaregression analysis of factors affecting between-study heterogeneity.

Heterogeneity source	Coefficients	Std. err.	*P* value
Sample size	-0.0806024	.0698194	0.271

**Table 3:** Predictors of mothers' care-seeking behavior for common childhood illnesses in Ethiopia.

Determinants	Comparison	Number of studies	Sample size	OR (95% CI)	*P* value	*I* ^2^ (%)	Heterogeneity test (*P* value)
Knowledge	Good vs. poor	2	528	1.15 (0.45-3.08)	0.768	95.6	≤0.001
Age of mother	≤28 years vs. >28	2	777	0.87 (0.25-3.02)	0.838	90.4	0.001
Educational status	No education vs. other^1^	3	1522	0.33 (0.21-0.54)	≤ 0.001	48.9	0.141
Marital status	Single vs. other^2^	2	1329	0.35 (0.22-0.55)	≤ 0.001	0.0	0.804
Perceived severity	Yes vs. no	8	4200	0.47 (0.17-1.26)	0.135	97.0	≤0.001

Others: ^1^primary and secondary tertiary. Others: ^2^married, divorced, and widowed.

## Data Availability

The data used on this study is fully available with request.
